# Sperm Chromatin Status and DNA Fragmentation in Mouse Species with Divergent Mating Systems

**DOI:** 10.3390/ijms242115954

**Published:** 2023-11-03

**Authors:** Clara Agudo-Rios, Ana Sanchez-Rodriguez, Ingrid I. D. Idrovo, Juan Ángel Laborda-Gomariz, Ana J. Soler, Maria E. Teves, Eduardo R. S. Roldan

**Affiliations:** 1 Department of Biodiversity and Evolutionary Biology, Museo Nacional de Ciencias Naturales (CSIC), 28006 Madrid, Spain; 2 SaBio IREC (CSIC-UCLM-JCCM), ETSIAM, Campus Universitario, 02071 Albacete, Spain; 3 Department of Obstetrics and Gynecology, Virginia Commonwealth University, Richmond, VA 23298, USA

**Keywords:** chromatin compaction, chromomycin A3, aniline, Diff-Quik, DNA fragmentation, SCSA

## Abstract

Sperm DNA integrity and chromatin status serve as pivotal indicators of sperm quality, given their intricate link to sperm function, embryo development, and overall fertility. Defects in chromatin compaction, which are often associated with compromised protamine content, can lead to damaged DNA strands. In this study, the chromatin status and possible correlation with DNA damage was assessed in males of three mouse species: *Mus musculus*, *M. spretus*, and *M. spicilegus*. We employed various staining methods, including aniline blue, methylene blue (Diff-Quik), toluidine blue, and chromomycin A3, to assess chromatin compaction in cauda epididymal sperm. Samples were also analyzed by the sperm chromatin structure assay (SCSA) to estimate DNA fragmentation (%tDFI, %HDS). Analyses were carried out on freshly collected sperm and cells incubated for 3 h in a HEPES-buffered modified Tyrode’s medium simulating conditions of the female reproductive tract. Notably, the analysis of chromatin status yielded minimal abnormal values across all three species employing diverse methodologies. SCSA analyses revealed distinct variations in %tDFI between species. Following sperm incubation, the percentages of sperm stained with methylene blue exhibited differences among the species and were significantly correlated to the DNA fragmentation index. HDS demonstrated correlations with the percentages of sperm stained by aniline blue, methylene blue, and chromomycin A3. Overall, chromatin compaction was high across all species, with limited differences among them. The relationship between chromatin status and DNA integrity appeared to be related to levels of sperm competition among species.

## 1. Introduction

The compaction of the paternal genome is necessary for optimal sperm functionality. A highly condensed sperm nucleus will improve sperm hydrodynamics, which translates into faster sperm movement and an advantage during oocyte fertilization [1,2]. In addition, chromatin compaction protects the paternal DNA from internal or external factors, such as nucleases or free radicals, which may cause damage to the genetic material [2,3,4,5]. DNA damage has been shown to affect sperm morphology, fertilization, and embryo development in various species [6,7,8]. Consequently, the integrity of sperm DNA assumes a pivotal role as a key indicator of sperm quality [5,9,10]. The maintenance of sperm DNA integrity may be under intense selective pressure with, in particular, a strong selection for protective mechanisms. However, comparative studies in rodents revealed that high levels of postcopulatory sexual selection (sperm competition) is associated with increased DNA damage [11], perhaps as a cost of the evolution of more competitive sperm with enhanced metabolism and high performance.

DNA packaging and chromatin compaction function differently in sperm cells than in somatic cells. During spermatogenesis, chromatin undergoes a series of structural and functional changes and evolves from a highly functional and genetically active state (similar to that observed in somatic cells) to a deprogrammed and quiescent state, necessary for the silencing of gene groups that will be reactivated after fertilization [12].

The DNA structure within somatic cells is associated with nucleosomal complexes formed through the binding of histones to DNA strands, as a result of the abundance of positively charged lysines and arginines that bind to the negatively charged double helix [13]. During the post-meiotic phase of spermatogenesis, i.e., spermiogenesis, histones are replaced by transition nuclear proteins and protamines. Protamines are basic proteins rich in arginine and cysteine and they are responsible for the final packaging of DNA [2,12]. The high level of compaction is due to the covalent bonds that these protamines induce in chromatin, leading to the formation of structures known as toroids [14]. A small fraction of sperm DNA remains compacted by histones [1,15]. Thus, certain features of chromatin organization present in somatic cells are also found in sperm cells [16].

Abnormalities in protamines have been related to male infertility [17]. A deficiency in protamine levels [18], in the ratio of protamines 1 and 2 [19], in the number of cysteine groups in protamine 1 [20], or in the replacement of histones by protamines [1] may cause DNA fragmentation. This, in turn, may contribute to abnormalities in sperm morphology, reduced sperm motility, and decreased fertilization rates [3,21], or even result in mutations that compromise the survival of offspring [22].

Several methods exist for the evaluation of chromatin status, which may reflect protamination efficiency. They include staining with aniline blue, methylene blue, toluidine blue or chromomycin A3 [4,23]. Aniline blue staining allows for the identification of immature spermatozoa with histone retention, resulting from a defect in the substitution of this protein by protamines [1,24,25]. The dye reacts with lysine residues, which are abundant in histones. A dark blue staining of spermatozoa indicates high levels of histones [26]. Given that protamines are rich in arginine and cysteine, properly protaminated spermatozoa exhibit a pale blue or slightly pinkish color revealed by the eosin included in the staining method [25,27,28,29]. Methylene blue and its derivatives, which are components of the commercial kit Diff-Quik, bind to negatively charged proteins such as DNA [30,31]. They enable the identification of different levels of chromatin decondensation [32]. Toluidine blue is a metachromatic dye that binds to damaged DNA with available phosphate groups; meaning that the less dense the chromatin, the more intense the color generated by toluidine blue [3,33]. Finally, chromomycin A3 is a fluorochrome derived from the bacterium *Streptomyces cerevisiae*, which binds to guanine- and cytosine-rich sites and competes with protamines for binding to the minor groove of DNA [33,34]. Results obtained with this assay are considered a strong indicator of lack of chromatin condensation due to protamine deficiency [5,34,35,36]. Cells can be distinguished depending on whether they show bright fluorescence (in the entire head or in part) as cells with abnormal protamination, or low fluorescence indicative of normal protamination [26,37,38,39].

These methods have been used mainly for the assessment of protamination in human [5,18,21,25,29,40,41] and bovine [1,3,34,42,43,44] sperm. Some contradictory results have been reported using these methods in both species, especially with regard to associations with other sperm traits and fertility, which probably originate from the diversity of methods used. Studies in other species are limited, but some attention has been given to the mouse model to examine, for instance, the effect on chromatin status of anti-estrogens [45], estrogens [46], lack of antioxidant protection [47], defects in protamine expression [48], or protamine transport into the nucleus [49].

Before fertilization, spermatozoa need to survive for hours or days in the female tract [50,51], a period in which DNA damage may also occur. During this time, sperm are transported passively or swim actively to negotiate several barriers, and then experience changes necessary to acquire the ability to engage in fertilization [52]. The conditions that ensure survival and the acquisition of fertilizing capacity may be mimicked in vitro to assess the effects on sperm parameters, including DNA integrity. Comparative studies among mammals showed DNA damage in sperm from several species after incubation in vitro for several hours, which may relate to the varying levels of protection afforded by protamines [9]. In contrast, studies in rodents revealed no major effects on DNA integrity during incubation [11] although, under similar conditions of incubation, decreases in sperm motility, cell integrity, swimming descriptors, or ATP levels were noted [53]. This suggests that rodent sperm DNA may not be susceptible to negative external or internal factors during preparation for fertilization in the female tract. Alternatively, rodent sperm may experience high efficiency in protamination and, hence, tight chromatin compaction that would protect sperm DNA from damage.

The aim of this study was to examine sperm chromatin status in three mouse species (*Mus musculus*, *M. spretus*, and *M. spicilegus*) that exhibit differences in patterns of sperm formation, morphology, and function. A series of methods were employed to assess chromatin maturation, compaction, stability, and deficiency. In parallel, DNA fragmentation was assessed using the sperm chromatin structure assay (SCSA). Using this method, DNA integrity was quantified as the total DNA fragmentation index (tDFI), which is defined as the percentage of sperm cells with DFI > 25, and as the percentage of immature sperm cells, which are defined by a High DNA Stainability (HDS) value that corresponds to sperm with high green fluorescence. Possible relationships between chromatin status and DNA integrity were also examined. Finally, we studied if DNA integrity and chromatin status were affected by conditions that mimic a period of residence in the female tract through in vitro incubation of spermatozoa.

## 2. Results

### 2.1. Analysis of Sperm Chromatin Status

**Figure 1:**
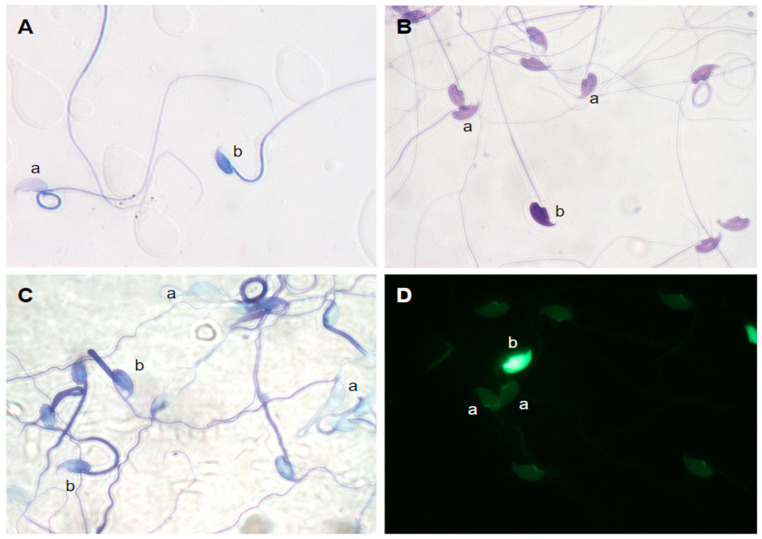
Spermatozoa from *Mus spretus* stained with (**A**) aniline blue, a: spermatozoon with mature chromatin (light blue or pink), b: spermatozoon with immature chromatin (dark); (**B**) Diff-Quik, a: spermatozoa with normally compacted chromatin (light violet), b: spermatozoon with abnormally compacted chromatin (dark violet); (**C**) toluidine blue, a: spermatozoa with normal chromatin (light blue), b: spermatozoa with damaged chromatin (dark blue/purple); (**D**) chromomycin A3, a: spermatozoa with correct protamination (dull green), b: spermatozoon with incorrect protamination and completely stained head (bright green).

Several staining methods were used to examine different aspects of sperm chromatin status (Figure 1). Aniline blue was used to assess sperm chromatin maturation, methylene blue (Diff-Quik) revealed chromatin compaction, toluidine blue reported chromatin stability and, finally, chromomycin A3 represented chromatin protamine deficiency. Spermatozoa were examined fresh, that is, upon collection from the cauda epididymis, and after a period of incubation for 3 h in a HEPES-buffered modified Tyrode’s (mT-H), which mimics a period of time spent by sperm cells in the female reproductive tract.

#### 2.1.1. Chromatin Maturation

Aniline blue staining distinguishes between sperm cells with dark blue (high histone levels) and light blue (high protamine levels) staining (Figure 1A).

The highest percentage of dark blue-stained cells was found in *M. musculus* (0.55 ± 0.23%) and *M. spretus* (0.55 ± 0.36%) (Table 1). No dark blue staining was found in *M. spicilegus* spermatozoa (0.00 ± 0.00%) in freshly collected samples. The percentage of dark blue-stained cells increased after a 3 h incubation period in all the species examined (1.45 ± 0.22%; 0.90 ± 0.32%, and 0.65 ± 0.26% for *M. musculus*, *M. spretus*, and *M. spicilegus*, respectively). The degree of change in the percentage of staining during incubation was obtained by calculating a new value, Δ3 h–0 h. Neither the absolute values nor this new variable showed significant differences after the incubation period. Differences between the species were also not significant. 

#### 2.1.2. Chromatin Compaction

Staining with the Diff-Quik kit includes a fixative solution with methanol, an eosin solution (which stains basic or positively charged proteins red), and a solution of methylene blue and derivatives, such as Azure B, which stains DNA blue. A sperm nucleus with normal chromatin compaction will show light staining, whereas abnormally compacted chromatin will exhibit dark staining (Figure 1B).

Consistently with aniline blue results, the highest percentage of abnormal chromatin compaction corresponded to *M. musculus* (1.15 ± 0.06%), followed by *M. spretus* (0.70 ± 0.20%) and *M. spicilegus* (0.50 ± 0.16%) (Table 2), but the differences did not reach statistical significance. After incubation of the samples for 3 h in mT-H medium, significant increases were observed in all three species, with percentages of 2.25 ± 0.14% (*p* = 0.03); 1.95 ± 0.59% (*p* = 0.01), and 1.75 ± 0.47% (*p* = 0.01), for *M. musculus*, *M. spretus*, and *M. spicilegus*, respectively. The increases observed over time (as revealed by Δ3 h–0 h) were not significantly different among species. 

#### 2.1.3. Chromatin Stability

Toluidine blue staining distinguishes cells with different levels of chromatin stability. With abnormal compaction, more DNA phosphate groups are exposed, and dark blue or violet staining is observed. If chromatin is compacted normally, the sperm will be stained light blue (Figure 1C).

Results obtained with this test differed considerably from those obtained with the two preceding methods. In the literature, most authors distinguish between stained and unstained cells for quantification, despite the wide staining spectrum ranging from light blue/green to dark blue/purple [3,33,54,55,56]. We found difficulties in the visualization of stained spermatozoa; there was a wide variation in staining patterns and it was not easy to appreciate a clear difference between stained and unstained spermatozoa. Because of the difficulties we encountered with the use of this method, consistent quantifications were not possible.

#### 2.1.4. Protamine Deficiency

Chromomycin A3 (CMA3) distinguishes different types of sperm cells depending on their staining patterns (Figure 1D, Table 3). Fully stained heads or partially stained heads, both with bright green fluorescence, are considered to have abnormal protamination (CMA3+). Sperm heads with dull green fluorescence are considered to have normal protamination (CMA3−).

In freshly collected sperm cells (no incubation, 0 h), the highest percentage of total abnormal protamination was found in *M. spretus* (1.20 ± 0.40%), with lower values in *M. musculus* (1.05 ± 0.18%) and *M. spicilegus* (0.40 ± 0.19%). After incubation of spermatozoa for 3 h in mT-H medium, the results obtained were 2.05 ± 0.34%; 1.80 ± 0.70%, and 1.95 ± 0.40%, respectively. A significant increase in total stained heads (complete plus partially stained) was observed in *M. spicilegus* after 3 h incubation (*p* = 0.04). As observed in previous assays, Δ3 h–0 h presents the same increase across species and no significant differences were found between them.

### 2.2. Analysis of DNA Fragmentation

DNA fragmentation was analyzed using the sperm chromatin structure assay (SCSA). Values of DNA fragmentation in freshly collected epididymal sperm (0 h), expressed as %tDFI (% high fragmentation index + % moderate fragmentation index), were 1.38 ± 0.14% for *M. musculus*; 0.56 ± 0.06% for *M. spretus*, and 0.35 ± 0.03% for *M. spicilegus*. After incubation for 3 h in mT-H medium, values were 1.89 ± 0.19%; 0.76 ± 0.17%; and 0.41 ± 0.05%, respectively (Table 4). Although the three species experienced an increase in %tDFI after incubation for 3 h, the result was only statistically significant for *M. musculus* (*p* = 0.0126). Among species, *M. musculus* had significantly higher %tDFI than the other two species (*p* < 0.0001 versus *M. spicilegus* at 0 h and 3 h; *p* = 0.0002 versus *M. spretus* at 0 h; *p* < 0.0001 versus *M. spretus* at 3 h) (Table 4).

We quantified %HDS values at 0 h and 3 h after incubation in mT-H (Table 4). The range was from 2.60 ± 0.25 in *M. spretus* to 3.77 ± 0.51 in *M. musculus* in freshly collected cells (0 h), and from 6.86 ± 2.58 in *M. spicilegus* to 7.84 ± 2.1 in *M. spretus* after 3 h of incubation in mT-H. No significant differences were found in %HDS among species in freshly collected cells (0 h) or after incubation (3 h). The increases in %tDFI or %HDS between 0 and 3 h of incubation (∆3 h–0 h) were not significantly different among species. 

### 2.3. Correlation between Chromatin Status and DNA Fragmentation

To examine possible associations between protamination (chromatin status) and DNA damage, we analyzed the percentages of spermatozoa with abnormal chromatin, as revealed by aniline blue, Diff-Quik, and CMA3 staining, in relation to values of DNA fragmentation quantified as total DFI and HDS after SCSA. Analyses were carried out for freshly collected cells (0 h before incubation in mT-H medium) and after 3 h of incubation in this medium. The differences observed at the end of incubation in relation to values before incubation (Δ3 h–0 h), both in chromatin status and DNA fragmentation, were also analyzed.

Total DFI values were not significantly related to values of chromatin compaction measured with aniline blue or CMA3, after either 0 or 3 h of incubation, when the three species were considered together (Table 5). A significant positive relationship was found in fresh (0 h) spermatozoa when chromatin status was assessed with Diff-Quik (r = 0.6163, *p* = 0.014) (Table 5). When species were considered separately, no significant relationships were found between DFI and chromatin compaction values in fresh sperm (0 h) or after 3 h of incubation (Table 5). 

**Figure 2:**
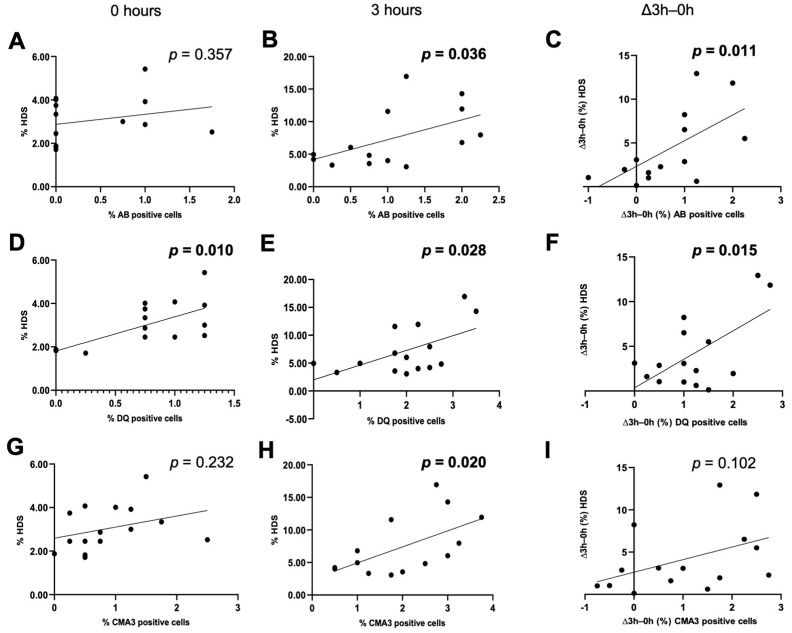
Relationships between DNA damage and chromatin status in freshly collected sperm and after 3 h of incubation in mT-H medium. Linear regressions between values of %HDS and % positive cells in each staining assay are plotted for all the species (*n* = 5 for each species). (**A**) Aniline blue in fresh sperm (0 h). (**B**) Aniline blue after 3 h of incubation. (**C**) Aniline blue Δ3 h–0 h. (**D**) Diff-Quik in fresh sperm (0 h). (**E**) Diff-Quik after 3 h of incubation. (**F**) Diff-Quik Δ3 h–0 h. (**G**) CMA3 in fresh sperm (0 h). (**H**) CMA3 in sperm after 3 h of incubation. (**I**) CMA3 Δ3 h–0 h. Values with *p* < 0.05 were considered statistically significant (in bold). Abbreviations. AB: aniline blue, DQ: Diff-Quik, CMA3: chromomycin A3, HDS: high DNA stainability.

In contrast, values of HDS, an indicator of immature sperm, showed significant positive relationships with Diff-Quick staining cells at 0 h (r = 0.6412, *p* = 0.01), and with values of all three staining assays after 3 h of incubation when all species were considered together (aniline blue: r = 0.545, *p* = 0.036; Diff-Quick: r = 0.567, *p* = 0.028; CMA3: 0.5927, *p* = 0.02). A significantly positive relationship was also found in Δ3 h–0 h values between HDS and aniline blue (r = 0.6345; *p* = 0.011) and Diff-Quik (r = 0.6132; *p* = 0.015), but it did not reach significance for CMA3 (r = 0.4381; *p* = 0.102) (Table 5, Figure 2).

When species were analyzed separately, *M. musculus* showed a significant relationship between HDS and CMA3 staining (at 3 h: r = 0.9148, *p* = 0.03; Δ3 h–0 h: r = 0.8926, *p* = 0.042), *M. spretus* showed a significant relationship between HDS and aniline blue (Δ3 h–0 h: r = 0.9833, *p* = 0.003) and *M. spicilegus* showed a high correlation between HDS and Diff-Quick, but it did not reach significance (r = 0.8480, *p* = 0.07).

## 3. Discussion

Failures in sperm chromatin compaction are the likely causes of low sperm quality, decreased fertilization success, and impaired embryo development. Thus, molecular changes occurring in chromatin reorganization and remodeling during sperm differentiation will be important determinants of sperm competence. In this study, chromatin status and DNA integrity have been compared in three mouse species that exhibit differences in semen traits, which are likely the result of evolution under different selective pressures [11,53]. Our results revealed high levels of sperm protamination, which paralleled little damage in DNA in sperm collected from the cauda epididymis. In sperm exposed to in vitro incubation conditions that mimic a period of residence in the female reproductive tract, a slight increase in DNA damage was detected, with a concomitant, limited reduction in protamination levels. This suggests that although some damage to sperm in these species may occur during sperm formation or epididymal transit, some additional negative effects could arise during transit in the female tract.

We examined chromatin status employing different staining techniques. Such techniques are supposed to identify aspects of chromatin maturation, i.e., histone to protamine transition (aniline blue), compaction (methylene blue), or stability (toluidine blue) in addition to estimation of protamination deficiency (chromomycin A3). One of these staining methods (toluidine blue) did not render consistent results, as obtained in other studies [56,57,58]; hence, no further analyses could be carried out using this approach.

Our results in the three mouse species examined showed low proportions of darkly (or brightly) stained spermatozoa that were indicative of abnormal chromatin status. These results are not very different from those obtained in several studies on laboratory mouse strains. For example, we found a range of 0–0.5% darkly stained sperm with aniline blue in the three closely related mouse species, whereas laboratory mouse sperm exhibited ~5% of darkly stained sperm [59] although in some strains it reached 15–22% [57,58,60]. With methylene blue (as incorporated in the Diff-Quik kit), the three mouse species we examined showed 0.5–1.1% darkly stained spermatozoa; however, to the best of our knowledge, there are no studies using this dye to assess chromatin status in mouse sperm cells. Finally, the three mouse species examined here exhibited 0.4–1.2% positively stained sperm (either completely or partially stained) with chromomycin A3, indicative of defective protamination [61], as opposed to the laboratory mouse, which exhibited values of ~3% [48,57]. In contrast, in other studies, cauda epididymal mouse sperm stained with chromomycin A3 revealed higher values (~10–20% stained) [45,46,47]. The ability of these staining methods to identify alterations in chromatin status is demonstrated by higher percentages of stained mouse sperm under different conditions, such as treatment with estrogens (30%) [46], oxidative stress, age (30–70%) [47], and protamine 1 (90%) or protamine 2 deficiencies (30%) [48]. In comparison to other species, alterations in chromatin status in the mouse are rather low; studies of human, bull, boar, dog, and cat sperm revealed higher proportions of spermatozoa with defects in sperm chromatin compaction [39,42,55,62,63,64,65]. Perhaps chromatin compaction is more efficient in mouse spermatozoa or genetic checkpoints during spermiogenesis are stricter, resulting in more high-quality sperm.

The question that arises is whether the results obtained with various methods to assess chromatin status are similar. The different methods are based on different principles, recognizing different aspects of chromatin compaction (from histone replacement to dye intercalation in DNA). Our results revealed low percentages of sperm exhibiting abnormal chromatin compaction with all methods, so it is difficult to make clear comparisons. In experimental studies exploring the effects of a variety of external agents, results among different techniques were not very different [57,58] although some staining methods appeared to be more sensitive for detecting changes than others. In contrast, discrepancies have also been reported [66].

DNA fragmentation was examined using the sperm chromatin structure assay (SCSA), which returns a fragmentation index (DFI) and high DNA stainability (HDS). SCSA is a powerful method to distinguish small fragmentation differences in the mouse [67]. This is probably due to the use of high-throughput flow cytometry, which allows the analysis of thousands of cells in a short period of time. DNA fragmentation index showed differences between the three mouse species. *M. musculus* cauda epididymal sperm had significantly higher %tDFI than *M. spretus* and *M. spicilegus*. There was also a clear trend for higher HDS values in *M. musculus* in relation to the other two species. These results are consistent with our earlier findings revealing differences in DNA integrity in rodent spermatozoa [11].

Protamination and DNA integrity will both be determined during the processes of sperm differentiation and subsequent sperm maturation in transit along the epididymis [56,61]. In addition, during the period between ejaculation and interaction with oocytes, sperm will be exposed to an additional series of exogenous factors and will generate potentially detrimental endogenous factors. Of particular importance is the time spermatozoa spend in the female reproductive tract. To explore possible effects on chromatin status and DNA fragmentation after release from the male tract, spermatozoa were incubated under conditions that mimic reproductive tract fluids. After incubation for 3 h in a HEPES-containing modified Tyrode’s medium, the three species exhibited higher percentages of stained spermatozoa, which suggests some deterioration in chromatin status. Experiments carried out in the past showed that these incubation conditions lead to a decrease in several sperm parameters (motility, vitality, cell structural integrity, kinematics) [53,68].

Regarding the effects on DNA fragmentation during incubation, we observed a consistent trend for an increase in tDFI in all three species, but it was only significant for *M. musculus*. In addition, there was a trend towards an increase in HDS values but it did not reach significance. This was probably due to the minimal values in DNA damage observed in the different species before or after incubation. These results agree with our earlier study [11] in which we did not observe a substantial increase in sperm DNA damage post-incubation in rodent species. These findings reinforce the idea that major problems in DNA integrity occur before ejaculation in these species. In other animals, results vary, with DNA integrity being affected by incubation for some hours in some species but not in others [9]. The different DNA fragmentation rates among species may be an evolutionary outcome associated with nuclear remodeling/packaging rather than an actual defect [20].

Although our study may not be directly related to the diagnosis or treatment of male fertility, our results could serve to argue that chromatin status is important to protect DNA from endogenous or exogenous insult. Aberrations in DNA compaction by defects in protamination can cause DNA fragmentation and other alterations in seminal parameters, ultimately having clinical implications such as reduced male fertility and the onset of genetic mutations in offspring [1,22]. Protamination and sperm DNA fragmentation in human sperm has been assessed using various methods [3,5,7,42,69,70]. In bull sperm, positive correlations between DFI values and the percentage of spermatozoa with low protamine content, assessed with chromomycin A3, were found [42,63], although one study found no association between these parameters [71]. In cats, abnormal staining with methylene blue (Diff-Quik) has been found to correlate positively with sperm DNA defects detected using the TUNEL assay [62].

We were able to evaluate the relationship between protamination and DNA integrity with divergent mating systems and sperm competitiveness in different mouse species. Our results showed a positive and significant correlation between tDFI, which is a measure of DNA fragmentation, and proportion of darkly stained sperm with methylene blue (Diff-Quik), which is a measure of abnormal chromatin compaction. This association was found when fresh samples (not incubated in medium) from the three species were analyzed together. A similar positive correlation was found between %HDS, which measures DNA immaturity, and percentage of freshly collected sperm staining with methylene blue when all species were considered together. No significant relationships were noted when each species was examined separately. After incubation for 3 h, a significantly positive correlation was seen between %HDS and the percentage of sperm with deficient chromatin status after staining with aniline blue, Diff-Quik, or CMA3 when all species were considered together. The relationship was also seen for *M. musculus* when chromomycin A3 was employed. The lack of significant associations at the species level may relate to the low values of both parameters in the majority of cases. An increase in damage in relation to protamination status was also seen in a comparative study examining the possible effect of the number of cysteine residues of protamine [9].

Protamination status and DNA integrity, and the interaction between them, may be under some form of concerted selection. We have previously reported that there is a positive relationship between the intensity of sperm competition (as inferred from relative testes mass) and DNA damage in epididymal sperm [11]. It was suggested that the production of very competitive sperm (as with high sperm competition levels) may generate such a cost in DNA integrity [11]. Our present study was carried out with a small subset of rodent species that differ in sperm competition levels [53]. A clear trend was observed in DNA fragmentation levels in relation to sperm competition, which was lower in species with more competition (*M. spicilegus*) and higher in species with less competition (*M. musculus*). *M. spicilegus* also showed the lowest abnormal histone level, lowest chromatin compaction deficiency, highest protamination, generally followed by *M. spretus* and *M. musculus*. This represents the first evidence of an association between chromatin status and protamination in relation to DNA integrity in rodents, and the possible influence of sperm competition as a selective force underlying this association.

## 4. Materials and Methods

### 4.1. Reagents

Unless indicated otherwise, all reagents were purchased from Sigma-Aldrich Co. (Madrid, Spain).

### 4.2. Animals

Males were maintained in standard conditions with water and food (balanced feed supplemented with seeds) ad libitum, with a photoperiod of 14 h of light and 10 h of darkness, temperature of 22 °C, and relative humidity of 55–60%. Adult males (4–12 months old) of *Mus musculus* (*n* = 5), *M. spretus* (*n* = 5), and *M. spicilegus* (*n* = 5) were used for sperm collection and analyses.

The study was approved by the Review Board of the Museo Nacional de Ciencias Naturales (CSIC) and the Comunidad de Madrid (reference 28079-47-A). Animal handling was performed following the specific guidelines and standards of the Society for the Study of Reproduction for the use of experimental animals, and the Spanish Animal Protection Regulation RD53/2013, which is in accordance with the European Union Regulation 2010/63.

### 4.3. Sperm Collection

Animals were sacrificed by cervical dislocation. Males were dissected along the midline and the testes, vas deferens, and cauda epididymis were removed. The contents of the vas deferens were pushed with forceps towards the epididymal cauda before the latter was removed. Caudae were placed in a Petri dish containing a modified Tyrode’s solution (mT-H) with 20 mM HEPES, 5.56 mM D-glucose, 50 μg/mL kanamycin, 5 μg/mL phenol red, and 4 mg/mL bovine serum albumin (fatty acid-free), with an osmolality of 295 mOsm/kg and a pH of 7.4 at 37 °C, under air. Slight cuts were made in the caudae to allow spermatozoa to swim out to the medium. After 10 min of incubation at 37 °C, tissue was discarded and the samples were transferred to Eppendorf tubes.

### 4.4. Aniline Blue Staining

The protocol for staining was based on that of Franken et al. [72] as modified by Wong et al. [25]. Briefly, two smears with 10 μL of sample were prepared and allowed to air dry. They were then fixed with 500 μL per slide of 3% buffered glutaraldehyde in 0.2 M phosphate buffer for 30 min at room temperature. The slides were rinsed in distilled water and stained for 5 min with 5% aniline blue (Thermo Fisher Scientific Inc., Waltham, MA, USA) dissolved in 4% acetic acid (pH 3.5). The slides were then rinsed with water and stained with 0.5% eosin for 1 min. After air-drying, slides were mounted with DPX.

A total of 200 spermatozoa per slide were counted using an Eclipse E400 light microscope (Nikon, Tokyo, Japan) using oil immersion (100× objective). Two staining patterns were differentiated: light blue or pink (low histone level) and dark blue (high histone levels).

### 4.5. Diff-Quik Staining

The methodology used for the Diff-Quik kit (RAL Diagnostics, Martillac, France) followed Windt et al. [32] and Sousa et al. [30]. Two smears with 10 μL of sample were prepared and allowed to air dry. Then, they were immersed sequentially in fixative, solution I (eosin Y) and solution II (methylene blue) for 1 min, 25 s and 25 s, respectively, following the manufacturer’s instructions. After rinsing in distilled water and air drying, slides were mounted with DPX.

Smears were analyzed under a light microscope Nikon Eclipse E400 using oil immersion (100× objective). A total of 200 spermatozoa per slide were counted, making a distinction between light staining (normal chromatin status) and dark staining (abnormal chromatin status).

### 4.6. Toluidine Blue Staining

The method described by Krzanowska [56] for mice was used. Two smears of 10 μL of sample were prepared and air dried. The slides were then fixed in ethanol–acetic acid (3:1) for 2 min at room temperature. After drying, they were stained for 15 min in 1% toluidine blue in distilled water. After rinsing in distilled water and air drying, the smears were mounted with DPX before counting.

A total of 200 spermatozoa per slide were counted using a light microscope (Nikon Eclipse E400) using oil immersion (100× objective). Staining of spermatozoa was categorized into two groups, unstained/light blue staining (mature/normal chromatin) or dark blue staining (damaged/abnormal chromatin).

### 4.7. Chromomycin A3 Staining

The methodology used for chromomycin A3 staining was based on protocols by Lolis et al. [36] and Castro et al. [34]. Smears with 10 μL of sample were made and allowed to air dry. Then, each slide was fixed in ethanol:acetic acid (3:1) for 5 min at 4 °C and air dried. A volume of 100 μL of CMA3 (Thermo Fisher Scientific Inc., Waltham, MA, USA) solution at a concentration of 0.25 mg/mL in McIlvain buffer (7 mL of 0.1 M citric acid + 32.9 mL of 0.2 M Na_2_HPO_4_, pH 7.0, containing 10 mM MgCl_2_) and 1% DMSO (to facilitate dissolution of CMA3) was carefully placed on the slide, spread with a coverslip, and incubated for 20 min at room temperature in the dark. Slides were then rinsed in McIlvain buffer and mounted with a drop of buffered glycerol (0.2 M glycerol:phosphate buffer, 1:1) for microscopic analysis. 

A Nikon Eclipse E600 fluorescence microscope was used with appropriate filters (EX 400–440, DM 455, BA 470) and an oil immersion objective (100×). Two hundred spermatozoa per slide were counted, identifying those with normal protamination or chromatin compaction (dull green fluorescence) and those with partial or complete protamination or incorrect chromatin compaction (bright green fluorescence).

### 4.8. DNA Fragmentation Analysis

Sperm DNA fragmentation was examined using the sperm chromatin structure assay (SCSA) [11]. This assay evaluates the degree of DNA denaturation by measuring the metachromatic shift of acridine orange using flow cytometry. The assay includes the incubation of spermatozoa with an acidic compound to allow for the denaturation of sperm DNA and expose DNA breaks. Acridine orange binds to DNA and emits green fluorescence when it binds to double-stranded DNA and red fluorescence when it binds to single-stranded DNA (indicating that the DNA is fragmented in that area). The DNA fragmentation index (DFI) was calculated as the ratio of red fluorescence to total (red plus green) fluorescence. Spermatozoa with high DFI have damaged DNA. High DNA stainability (HDS) was quantified as representative of retained nuclear histones, which is indicative of immature sperm.

Aliquots of sperm suspensions were snap-frozen immediately after release from the epididymis and 3 h after incubation in mT-H, and then stored at −80 °C until analysis. After thawing, samples were diluted with TNE buffer (0.15 M NaCl, 0.01 M Tris-HCl, 1 mM EDTA; pH 7.4) at a final concentration of 2 × 10^6^ sperm/mL. A volume of 200 μL of each sample was mixed with 400 μL of an acid-detergent solution (0.08 N HCl, 0.15 M NaCl, 0.1% Triton X-100; pH 1.4) for 30 s. Then, 1.2 mL of acridine orange staining solution (0.1 M citric acid, 0.126 M Na_2_HPO_4_, 0.0011 M disodium EDTA, 0.15 M NaCl; pH 6.0, 4 °C) containing 6 µg/mL electrophoretically purified acridine orange was added. For flow cytometry, an argon laser (488 nm) was used to excite acridine orange. DNA denaturation was indicated as total DNA fragmentation index (tDFI), defined as the percentage of spermatozoa with a DFI value over 25. Percentage of immature spermatozoa was defined as High DNA Stainability value (HDS), which is the percentage of spermatozoa with green fluorescence higher than channel 600 of 1024 channels.

### 4.9. Statistical Analyses

Statistical analyses were performed with GraphPad Prism 8.0 software (GraphPad, San Diego, CA, USA). Data were normally distributed as revealed by tests carried out with this software, namely Anderson–Darling, D’Agostino, Shapiro–Wilk, and Kolmogorov–Smirnov tests. Two-tailed, two-way analysis of variance (ANOVA) was performed, followed by Tukey’s multiple range test, to detect significant differences (*p* < 0.05). Pearson’s test was performed to evaluate the correlation coefficients (*r*) between fragmentation (tDFI or HDS) and the percentage of cells stained with each of the dyes used for evaluation of chromatin status. Correlations were regarded as significant when *p* < 0.05. Data are presented as mean ± standard error of the mean (SEM). 

## 5. Conclusions

The evaluation of chromatin status as an additional parameter of sperm quality has been strongly emphasized in recent years. Our study suggests that protamines, histones, and chromatin compaction appear to be maintained across rodents, and that incubation does not appear to be a key factor for the worsening of these parameters in most of these species. In addition, sperm competition may have a role in chromatin status and DNA damage, but additional studies are required. The effect of abnormal chromatin compaction on fertility parameters in rodents still needs to be demonstrated. The different studies do not yet allow the determination of whether chromatin status has a strong impact on fertility prediction, but most of them indicate that these techniques should be added as another parameter of sperm quality assessment.

## Figures and Tables

**Table 1:** Percentage of dark blue-stained cells observed using aniline blue. Staining was quantified soon after sperm collection from epididymis (0 h) and after incubation in mT-H medium (3 h). Differences between values at the end and at the beginning of incubations were calculated (∆3 h-0 h). Data are means ± SEM (*n* = 5 for each species) and were analyzed using a two-tailed, two-way ANOVA. Values with *p* < 0.05 were considered significant.

Species	Percentage of Stained Cells at			
	0 h	3 h	Δ3 h–0 h	
*M. musculus*	0.55 ± 0.23	1.45 ± 0.42	0.90 ± 0.39	
*M. spretus*	0.55 ± 0.36	0.90 ± 0.32	0.35 ± 0.52	
*M. spicilegus*	0.00 ± 0.00	0.65 ± 0.26	0.65 ± 0.26	

**Table 2:** Percentage of dark-stained cells observed using Diff-Quik. Staining was recorded in freshly collected cells (0 h) and after 3 h of incubation in mT-H medium. Differences between values at the end and at the beginning of incubations were calculated (∆3 h–0 h). Data are presented as mean ± SEM (*n* = 5 for each species) and were analyzed using a two-tailed, two-way ANOVA. Values with *p* < 0.05 were considered significant. Same letters in the same row indicate significant differences between time points.

Species	Percentage of Stained Cells at		
	0 h	3 h	Δ3 h–0 h
*M. musculus*	1.15 ± 0.06 ^a^	2.25 ± 0.14 ^a^	1.10 ± 0.19
*M. spretus*	0.70 ± 0.20 ^a^	1.95 ± 0.59 ^a^	1.25 ± 0.50
*M. spicilegus*	0.50 ± 0.16 ^a^	1.75 ± 0.47 ^a^	1.25 ± 0.36

**Table 3:** Percentage of positively stained cells observed using CMA3 assay. Three groups were considered according to staining patterns: completely stained head, partially stained head, and total number of stained cells (complete + partial). Staining was quantified in freshly collected cells (0 h) and after 3 h of incubation in mT-H medium. Differences between values at the end and at the beginning of incubation were calculated (∆3 h–0 h). Data are presented as mean ± SEM (*n* = 5 for each species) and were analyzed using a two-tailed, two-way ANOVA. Values with *p* < 0.05 were considered significant. Same letters in same rows indicate significant differences between time points.

	Completely Stained (%)		Partially Stained (%)		Total Staining (%)		Total Staining
Species	0 h	3 h	0 h	3 h	0 h	3 h	Δ3 h–0 h
*M. musculus*	0.95 ± 0.20	1.40 ± 0.47	0.10 ± 0.06	0.40 ± 0.29	1.05 ± 0.18	1.80 ± 0.70	0.75 ± 0.68
*M. spretus*	1.15 ± 0.36	1.80 ± 0.24	0.05 ± 0.05	0.25 ± 0.19	1.20 ± 0.40	2.05 ± 0.34	0.85 ± 0.56
*M. spicilegus*	0.35 ± 0.17	1.35 ± 0.33	0.05 ± 0.05	0.60 ± 0.15	0.40 ± 0.19 ^a^	1.95 ± 0.40 ^a^	1.55 ± 0.35

**Table 4:** Percentage of two SCSA parameters of DNA fragmentation in freshly collected sperm (0 h) and after 3 h of incubation in mT-H medium. Differences between values at the end and at the beginning of incubations were calculated (∆3 h–0 h). Data are presented as mean ± SEM (*n* = 5 for each species) and were analyzed using a two-tailed, two-way ANOVA. Values with *p* < 0.05 were considered significant. Same letters in the same row indicate significant differences between time points. Same numbers in the same column indicate significant differences between species at a given time. Abbreviations: tDFI: total DNA fragmentation index; HDS: high DNA stainability; see Section 4 for explanation of these parameters.

Species	Parameter	0 h	3 h	Δ3 h–0 h
*M. musculus*	%tDFI	1.38 ± 0.14 ^a,1,2^	1.89 ± 0.19 ^a,1,2^	0.51 ± 0.22
	%HDS	3.77 ± 0.51	6.98 ± 1.45	3.21 ± 1.24
*M. spretus*	%tDFI	0.56 ± 0.06 ^1^	0.76 ± 0.17 ^1^	0.19 ± 0.12
	%HDS	2.60 ± 0.25	7.84 ± 2.14	5.24 ± 2.07
*M. spicilegus*	%tDFI	0.35 ± 0.03 ^2^	0.41 ± 0.05 ^2^	0.06 ± 0.05
	%HDS	2.76 ± 0.48	6.86 ± 2.58	4.11 ± 2.24

**Table 5:** Correlation between chromatin compaction (% positive staining) evaluated with different assays and DNA fragmentation (% tDFI and % HDS; see Material and Methods for explanation of these parameters) at 0 h and after 3 h of incubation in mT-H medium. Pearson’s test was used for analyses. Values with *p* < 0.05 were considered statistically significant and are shown in bold. Abbreviations. AB: aniline blue, DQ: Diff-Quik, CMA3: chromomycin A3, tDFI: total DNA fragmentation index, HDS: high DNA stainability. Values are *r* coefficients with *p* values in parentheses (*n* = 5 for each species). Since aniline blue values at 0 h for *M. spicilegus* were 0%, a correlation could not be calculated for this parameter.

Species	Parameter	tDFI			HDS		
		0 h	3 h	Δ3 h–0 h	0 h	3 h	Δ3 h–0 h
All species	AB	0.2925 (0.290)	0.3679 (0.177)	0.1002 (0.722)	0.2562 (0.357)	**0.5450** **(0.036)**	**0.6345** **(0.011)**
	DQ	**0.6163** **(0.014)**	0.2625 (0.345)	0.0486 (0.864)	**0.6412** **(0.010)**	**0.5670** **(0.028)**	**0.6132** **(0.015)**
	CMA3	0.2918 (0.291)	0.0382 (0.892)	−0.0833 (0.768)	0.3284 (0.232)	**0.5927** **(0.020)**	0.4381 (0.102)
*M. musculus*	AB	0.2858 (0.641)	−0.2360 (0.702)	−0.0253 (0.968)	0.5228 (0.366)	0.7169 (0.173)	0.7819 (0.118)
	DQ	0.4016 (0.503)	0.6120 (0.273)	0.4455 (0.452)	0.4116 (0.491)	−0.0899 (0.886)	−0.0478 (0.939)
	CMA3	0.5115 (0.378)	0.4793 (0.412)	0.3814 (0.526)	0.4551 (0.441)	**0.9148** **(0.030)**	**0.8926** **(0.042)**
*M. spretus*	AB	−0.3486 (0.565)	0.1641 (0.792)	−0.0292 (0.963)	0.0717 (0.909)	0.8115 (0.095)	**0.9833** **(0.003)**
	DQ	0.0602 (0.923)	0.2023 (0.744)	−0.0995 (0.874)	0.5443 (0.343)	0.5308 (0.357)	0.6044 (0.280)
	CMA3	−0.0890 (0.887)	0.0645 (0.918)	−0.510 (0.379)	0.4174 (0.485)	0.4768 (0.417)	0.5130 (0.377)
*M. spicilegus*	AB	-	−0.1220 (0.845)	0.4029 (0.501)	-	0.4940 (0.398)	0.4454 (0.452)
	DQ	−0.8715 (0.054)	−0.2552 (0.679)	0.3730 (0.536)	0.7893 (0.112)	0.8107 (0.096)	0.8480 (0.070)
	CMA3	−0.5783 (0.307)	−0.3926 (0.513)	−0.3614 (0.550)	0.5568 (0.330)	0.5962 (0.289)	0.1483 (0.812)

## Data Availability

The raw data supporting the conclusions of this article will be made available by the authors without undue reservation.
